# Laparoscopic surgery for broad ligament adenomyoma: A case report and literature review

**DOI:** 10.1097/MD.0000000000043399

**Published:** 2025-07-11

**Authors:** Siman Liu, Xiu Lin, Yujie Huang, Zhong Lin

**Affiliations:** a Department of Gynecology, Guangxi Zhuang Autonomous Region Reproductive Hospital, Nanning, Guangxi, China.

**Keywords:** adenomyoma, broad ligament, laparoscopy

## Abstract

**Rationale:**

Adenomyoma of the broad ligament is rare. Owing to the absence of typical clinical signs and imaging methods, preoperative diagnosis is challenging, and it is mainly diagnosed through surgical pathology.

**Patient concerns:**

The patient experienced recurrent lower abdominal pain for 4 years. Treatments with traditional Chinese medicine and anti-inflammatory drugs were ineffective, and painkillers became less effective over time owing to resistance. Multiple ultrasounds revealed a mass in the right adnexa, but gynecological tumor markers were normal.

**Diagnosis:**

The preoperative diagnosis was unclear, but a mass in the right adnexa was suspected.

**Interventions:**

The patient underwent laparoscopic exploration, which revealed a 25 mm × 25 mm × 20 mm spherical mass on the anterior leaf of the right broad ligament. It had clear boundaries, a hard texture, and was not attached to the uterus, fallopian tube, or ovary. The mass was completely excised. Gross examination of the specimen revealed a disorganized cut surface with a small amount of coffee-colored fluid. Postoperative pathology confirmed it was an adenomyoma of the broad ligament.

**Outcomes:**

The patient’s lower abdominal pain resolved after surgery, and follow-up has shown no recurrence of pelvic masses.

**Lessons:**

Adenomyoma of the broad ligament can cause recurring abdominal pain. Preoperative diagnosis is challenging, but laparoscopic surgery can confirm the diagnosis, remove the tumor, and relieve symptoms.

## 1. Introduction

Uterine adenomyoma is a benign, estrogen-dependent uterine lesion that typically occurs in women of childbearing age. Common clinical symptoms include secondary dysmenorrhea, heavy menstrual bleeding, anemia, uterine enlargement, and infertility. Treatment options include medication and surgery. Hysterectomy is an effective radical treatment for uterine adenomyoma, but it is unacceptable for women who wish to retain their fertility. In these cases, laparoscopic enucleation of the lesion is usually preferred. In recent years, high-intensity focused ultrasound (HIFU) has emerged as a noninvasive treatment that can reduce tumor size and alleviate dysmenorrhea.^[1,2]^ Studies have also shown that changes in the levels of Carbohydrate Antigen 199, Carbohydrate Antigen 125 (CA125), Matrix Metalloproteinase-9, and Matrix Metalloproteinase-2 may be associated with the recurrence of uterine adenomyoma.^[3]^ Adenomyoma of the broad ligament is a type of extrauterine adenomyoma, which is relatively rare in clinical practice. It is typically located in the pararectal space, ovary, peritoneum, or broad ligament.^[4]^ To date, there are no established epidemiological data regarding its incidence, with existing literature limited to isolated case reports. The pathogenesis of this condition remains poorly understood. This article presents a case of broad ligament adenomyoma managed successfully through laparoscopic surgery.

## 2. Case presentation

**Figure 1.**
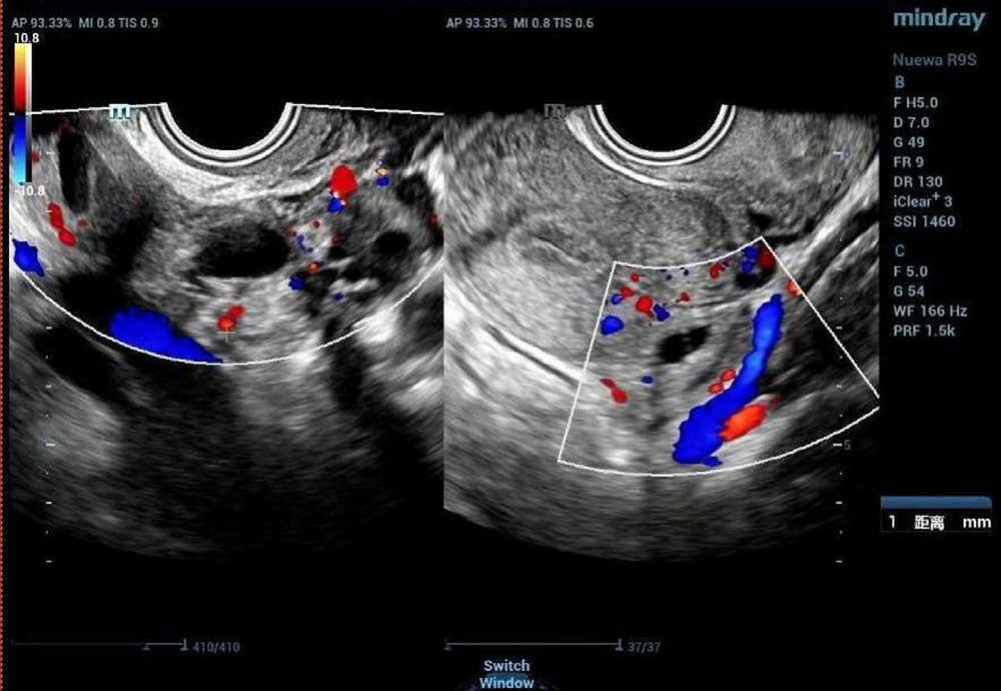
The ultrasound showed a mixed mass in the right adnexa.

The patient, a 29-year-old unmarried, nulliparous woman, was admitted to the hospital due to “recurrent dull lower abdominal pain for 4 years, which had worsened over the past year.” Four years ago, she began experiencing periodic dull pain in her lower abdomen, usually during her menstrual period. An ultrasound at an outside hospital revealed a mixed mass in the right adnexa, approximately 22 mm in diameter, with unclear nature. Gynecological tumor marker tests, including CA199, CA125, Carbohydrate Antigen 153, Carcinoembryonic Antigen, Alpha-Fetoprotein, and Human Epididymis Protein 4, showed no abnormalities. Follow-up ultrasounds of the adnexal mass revealed no changes. She had undergone multiple treatments with traditional Chinese medicine at other hospitals, but these were ineffective, and she required oral painkillers for pain relief. Over the past year, the lower abdominal pain had worsened, was no longer related to her menstrual cycle, and could last up to a month. She had been treated for “pelvic inflammatory disease” at another hospital, but the abdominal pain persisted, and painkillers were no longer effective. Upon admission, her CA125 level was 15.82 U/ml. Gynecological ultrasound showed a mixed-echo mass in the right adnexa, measuring 28 mm × 28 mm × 22 mm, with an unclear nature (Fig. 1).

**Figure 2.**
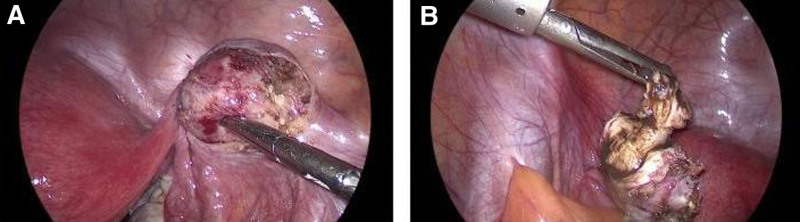
(A) The laparoscopy showed the adenomyoma of the right broad ligament. (B) Gross examination of the broad ligament tumor revealed a disorganized cut surface with a small amount of coffee-colored fluid.

Subsequently, a laparoscopic exploration was performed. During surgery, a spherical mass measuring 25 mm × 25 mm × 20 mm was found on the anterior leaf of the right broad ligament (Fig. 2A). The mass had clear borders, was hard in texture, and was not connected to the uterus, fallopian tubes, or ovaries. The mass was completely dissected and removed. Upon removal, the cut surface of the specimen revealed a disorganized structure with a small amount of coffee-colored fluid (Fig. 2B). The patient was discharged on the second day after surgery. Postoperative pathology confirmed the diagnosis of adenomyoma of the broad ligament. At the 6-month postoperative follow-up, the patient reported no abdominal pain, and no recurrence of the tumor was seen on ultrasound reexamination.

## 3. Discussion

Extrauterine adenomyomas are a rare condition. They may be asymptomatic, but when symptoms occur, they can include abdominal masses, menstrual changes, dysmenorrhea, urinary tract or rectal compression, and low back pain.^[4,5]^ Due to the lack of typical clinical features and imaging methods, preoperative misdiagnosis rates are extremely high, reaching 95.45% to 100%.^[6,7]^ These adenomyomas are often misidentified as uterine tumors, pelvic masses, ovarian tumors, or adnexal masses.

Broad ligament adenomyomas are classified into true and false types.^[7,8]^ True adenomyomas are remnants of degenerated tissues or components of the broad ligament itself from embryonic development. Some theories suggest they may arise from primordial germ cells that differentiate into endometrial tissue. If these cells are blocked or migrate improperly, they may form endometriosis and later develop into extrauterine adenomyomas. False adenomyomas originate from nearby organs like the uterus, fallopian tubes, or ovaries, or from malignant metastasis into the broad ligament. True adenomyomas of the broad ligament are strictly located within the ligament and are not connected to the ovaries, fallopian tubes, or uterus. The key to imaging differentiation is identifying the ovaries and uterus to rule out lesions originating from these structures.^[8,9]^

Adenomyoma of the broad ligament is a benign tumor, with diagnosis primarily established through postoperative histopathological examination. Microscopically, it is composed of endometrial glands and stroma intermixed with proliferative smooth muscle, without cellular atypia. The histology of broad ligament adenomyoma is dominated by smooth muscle components with scattered endometrial glands and stroma. It can be easily confused with smooth muscle-containing endometriosis and leiomyomas associated with endometriosis. In smooth muscle-containing endometriosis, smooth muscle metaplasia is typically focal, whereas adenomyomas predominantly consist of smooth muscle tissue. In leiomyomas associated with endometriosis, the endometriotic cysts are usually located at the periphery and are separate from the smooth muscle component. Immunohistochemical staining shows that the endometrial glandular and stromal cells express estrogen receptors and progesterone receptors, the stromal cells express Cluster of Differentiation 10, and the smooth muscle cells express Smooth Muscle Actin.^[10,11]^ Surgery is the main treatment, and the tumor is usually removed through surgical intervention.

This article reports a case of broad ligament adenomyoma. Follow-up ultrasounds after surgery have shown no recurrence of the tumor. Given that the broad ligament is rich in blood vessels and located near the ureter, care must be taken to avoid damaging the ureter during surgery. Proper hemostasis and closure of the tumor cavity are essential to prevent hematoma formation. Additionally, since the obturator nerve runs close to the inner surface of the pelvic wall, the use of electrocoagulation should be minimized to avoid nerve damage. Wenwen et al^[7]^ reported 2 cases of obturator nerve injury caused by electrocoagulation for hemostasis during broad ligament tumor surgery, resulting in reduced skin sensation or limited movement of the affected lower limb.

Yiran et al^[12]^ reported that 10 patients with broad ligament fibroids had good results with HIFU treatment, with a clinical tumor shrinkage rate of 9/10. However, due to the unique location of these adenomyomas – close to the intestines, ureters, ovaries, and pelvic blood vessels – there is a high risk of damaging surrounding tissues and organs during treatment. Additionally, preoperative diagnosis of broad ligament adenomyomas is very challenging, creating uncertainty for nonsurgical treatments. While the risk of malignant transformation in these adenomyomas is low, it cannot be completely ruled out. Torres et al^[10]^ reported a case of clear cell carcinoma-like changes in a broad ligament adenomyoma. Therefore, when using minimally invasive surgery to resect the tumor via rotary cutting, care should be taken to place the tumor in a specimen bag to prevent implantation and spread of the tumor in the abdominal cavity.

## 4. Conclusion

Adenomyoma of the broad ligament is rare in clinical practice, and preoperative diagnosis is challenging. It is usually a benign lesion, and surgery is the main treatment to remove the tumor. However, there is a very low risk of malignant transformation, so it is important to ensure a tumor-free operation during surgery.

## Acknowledgments

The authors thank the patient who participated in the present study for providing written permission to publish this case report.

## Author contributions

**Conceptualization:** Siman Liu, Xiu Lin, Yujie Huang.

**Data curation:** Siman Liu, Xiu Lin, Yujie Huang, Zhong Lin.

**Formal analysis:** Siman Liu, Xiu Lin, Yujie Huang, Zhong Lin.

**Writing – original draft:** Siman Liu.

**Writing – review & editing:** Siman Liu, Xiu Lin.
